# Changes in the Flavor of Cold-Pressed Tiger Nut Oil (*Cyperus esculentus* L.) During Refining Processes and Evaluation of Its Thermal Properties

**DOI:** 10.3390/foods14020301

**Published:** 2025-01-17

**Authors:** Yue Zhao, Yang Sun, Heyi Sun, Tianying Sun, Jian Ren, Chunli Song

**Affiliations:** 1 College of Food and Biological Engineering, Qiqihar University, Qiqihar 161006, China; qslzyy@126.com (Y.Z.);; 2 Engineering Research Center of Plant Food Processing Technology, Qiqihar University, Qiqihar 161006, China; 3 Key Laboratory of Agricultural Products Processing of Heilongjiang Province Ordinary University, Qiqihar University, Qiqihar 161006, China

**Keywords:** cold-pressed tiger nut oil, refining process, volatile compounds, thermal properties

## Abstract

Oil extracted from tiger nut is a good, edible source owing to its richness in unsaturated fatty acids. This study investigated the effects of the refining processes on the flavor components of crude tiger nut oil by GC-MS and focused on the thermal stability of the refined oil under high-temperature conditions. Three different refining processes were evaluated: citric acid-assisted hydration degumming, alkali deacidification and bleaching. In the present study, the neutralization refining resulted in 11.67% losses. The refined oil had higher brightness and transparency. Moreover, 109 volatiles were identified, mainly including aldehydes, alcohols, pyrazines and furans, the characteristic flavor compounds of which present a fatty, fresh and nutty flavor. Hence, the refining processes have a significant effect on the flavor components of tiger nut oil, and the accumulated information can be helpful in increasing the tiger nut oil quality to meet the market value. The results of the thermal properties indicated the significant degradation of oleic acid and linoleic acid with prolonged heating, leading to increases in the acid value by 17 times and the peroxide value by 31 times after prolonged heating at 210 °C for 10 h compared with those without heating. When the refined tiger nut oil was heated at 210 °C for 4 h, the carbonyl value (62.6 meq/kg) exceeded the recommended value, and after heating for 8 h, the total polar compound percentages (50%, the instrument limit value) also exceeded the national standard. In order to extend the cooking heating time, it is necessary to appropriately decrease the heating temperature. This study provides a scientific reference for the frying of tiger nut oil in food and the high-temperature treatment of food containing tiger nut oil.

## 1. Introduction

Tiger nut, as one of the major vegetable oil seeds, is known as groundnut, tiger nut fruit and yellow nutsedge [1,2]. It is now widely cultivated in tropical, subtropical and temperate regions of Europe, Africa, Asia and the Americas [3,4]. There are various nutrient contents of tiger nut, including high proportions of vitamin C and E (8–14 mg/100 g), protein (5–10%), dietary fiber (8–10%), starch (25–40%), oil (20–36%) and mineral elements (phosphorus, iron, potassium, etc.) [5,6,7]. It has been reported that tiger nut can be beneficial for decreasing some disease risks, such as cardiovascular disease and the formation of blood clots, associated with a lower risk of colon cancer [8,9]. Tiger nut has been shown to be beneficial to human health. At present, there are a few technological interests in and scientific reports about the extraction process, physicochemical properties, fatty acid composition and storage properties of edible oils. It has been reported that cold pressing can avoid the adverse effects of heating and organic solvents, which produce low yields of oil. A major advantage of cold pressing over other methods is that it is an environmentally friendly process and suitable for obtaining edible oils enriched with bioactive components [10,11]. Some studies have found that the fatty acid composition of tiger nut oil is similar to that of olive oil and camellia oil. It is a non-drying oil and has a lower degree of unsaturation [5]. A physicochemical analysis of tiger nut oil showed that the unsaturated fatty acids were rich in oleic acid and linoleic acid and it had a low iodine value [12,13]. Kim et al. analyzed the fatty acid composition of tiger nut oil and determined the fatty acid distribution of sn-1, sn-2 and sn-3 [14]. Therefore, it has better antioxidant properties.

At present, the research on tiger nut oil is mainly focused on the optimization of the extraction efficiency and the influence on the oil quality, and there is a lack of analysis on the volatile components of the refined oil. The effective analysis of the volatile aroma components in processing has an important guiding effect on the production of tiger nut oil with high nutritional value. Flavor is believed to be closely connected with the quantitative volatile compounds in oil, which is an important factor that affects the choices of numerous consumers [15,16]. It is of great significance to analyze the changes in the volatile aroma components in tiger nut oil during the refining process to reduce or even avoid the loss of specific aroma components and nutrients in the different refining processes.

Tiger nut oil could be widely used in the food industry, such as in fried food, biscuits and other food industries, as a substitute for other vegetable oils with its better antioxidant properties [5,17]. Frying is one of the traditional cooking methods in China, which is an important component of Chinese traditional food culture, and it is also a widely used food processing method in the food industry and home cooking. When frying with oil as the medium, through the application of high temperatures to mature food, in the heating process, the oil undergoes hydrolysis, oxidation, polymerization and other reactions to change its composition, and it then degrades into a volatile aldehyde, ketone and acid mixture. After heating at high temperatures, different edible oils will have different changes in their peroxide value, acid value, iodine value and other quality indicators, and the content and type of fatty acids will also change and even deteriorate. When they infiltrate into food, they are ingested by the human body. In the food industry of China, there are many kinds of oil commonly used for frying, but palm oil, soybean oil and hydrogenated soybean oil are mainly used. Studies have shown that consuming a large amount of palm oil increases the probability of cardiovascular disease, which is not good for human health [18]. However, soybean oil has a high degree of unsaturation, and triglycerides are prone to deterioration and poor stability in the frying process. Hydrogenated soybean oil forms large amounts of trans fatty acids during frying. Therefore, it is particularly important for human health to choose the right frying oil. Furthermore, abused frying oil is difficult to digest and can result in diarrhea and raise toxicological concerns.

At present, there are few research reports about the effects of the refining process on the flavor compositions of tiger nut oil or evaluations of thermal properties. Therefore, in this study, degumming, neutralization and bleaching were used to refine the tiger nut oil, and the detection and identification of key volatile compounds of different samples were analyzed by GC-MS, in order to provide the theoretical basis for the quality control and refining methods of tiger nut oil. At the same time, the thermal stability of oil plays a crucial role in its nutritional value. This paper studied the functional qualities of the refined tiger nut oil with high-temperature treatment and analyzed the thermal properties of the oil from the perspective of quality indicators. This study provides insights into the impacts of refining on flavor and thermal stability, which are critical for consumer acceptance and food industry applications. It provides some theoretical support for the further processing and utilization of the tiger nut oil and the development of high-value-added products.

## 2. Materials and Methods

### 2.1. Materials and Refining Treatment

The main materials used include citric acid (Tianjin Ruikai Chemical Reagent Co., Ltd., Tianjin, China); Trichloromethane and sodium hydroxide (NaOH) (Tianjin Maoda Chemical Reagent Co., Ltd., Tianjin, China); potassium hydroxide and acetic acid (Tianjin Tianli Chemical Reagent Co., Ltd., Tianjin, China); phenolphthalein indicator and sodium thiosulphate (Tianjin Kaitong Chemical reagent Co., Ltd., Tianjin, China); Wijs reagent, potassium iodide, soluble starch, cyclohexane and n-butanol (Tianjin Kemiou Chemical Reagent Co., Ltd., Tianjin, China).

Tiger nuts were purchased from a local factory in Daqing province (China). All of the cold-pressed tiger nut oil samples were self-made in our laboratory and stored at 4 °C under nitrogen. The conditions were as follows: The tiger nuts were cleaned, then crushed and the moisture adjusted to 8%. At room temperature, with the pressure 40~50 Mpa, the tiger nuts were pressed by automatic hydraulic press and then filtered to obtain the crude tiger nut oil.

Refining processes were carried out with slight modification [19]. The setting of key refining parameters was based on the preliminary results of our experimental team. Briefly, the first step was citric acid-assisted hydration degumming, in which the addition amount of citric acid was 0.25% (*w*/*w*), and the obtained oil was named DO. Then, neutralization was carried out by alkali refining and the obtained oil was marked as NO. Subsequently, activated earth was used as the adsorbent for bleaching and the obtained oil was marked as BO. The specific conditions of different refining processes are shown in Table 1.

The calculation formula of oil yield and loss rate in the refining process was as follows:

(1) $$\mathrm{Oil}\mathrm{yield}\;(\%)=\frac{\mathrm{oil}\mathrm{refined}\;(g)}{\mathrm{crude}\mathrm{oil}\mathrm{extracted}\;(g)}\times 100$$

(2) $$\mathrm{Oil}\mathrm{loss}\mathrm{rate}\;(\%)=(1-\frac{\mathrm{oil}\mathrm{refined}\;(g)}{\mathrm{refined}\mathrm{oil}\mathrm{in}\mathrm{last}\mathrm{process}\;(g)})\times 100$$

### 2.2. Measurement of Oil Color

ΔE* of the refined tiger nut oil was measured by Ultra Scan VIS with schema types: RSIN—specular reflection; light—CIELAB D65/10.

(3) $$\Delta E*=\sqrt{{\left(\Delta L\right)}^{2}+{\left(\Delta a\right)}^{2}+{\left(\Delta b\right)}^{2}}$$

### 2.3. Identification of Volatile Components by GC-MS

Headspace solid-phase microextraction (HS-SPME) [20]: The tiger nut oil weighed 1.5 g and was placed in a 20 mL headspace bottle. The extraction was carried out in a headspace vial with a CAR/PDMS fiber (film thickness 75 μm) at 80 °C for 20 min. The volatile components absorbed by the fiber were desorbed in the hot injection port of GC for 5 min at 250 °C.

Analysis of GC/MS [21]: GC/MS analysis was carried out using a full-scan system equipped with a TG-5 capillary column (30 m × 0.25 mm, 0.25 μm). The carrier gas was helium at a flow rate of 1.0 mL/min. The column was maintained at an initial temperature of 50 °C for 3 min and then programmed at 6 °C/min to 250 °C and maintained for 5 min. The interface temperature and the ion source temperature were set at 250 °C and 230 °C, respectively. The ionization mode was electron impact ionization mode with the electron energy 70 eV and a mass scanning range 40–650 amu.

Analysis of volatile components: The volatile compounds in tiger nut oil were separated by GC and identified by mass spectrometry. Each peak in the total ion current diagram was compared with the data from NIST and WILEY spectra. The peak area normalization method was used to quantitatively analyze the qualitative substances (match quality > 600). The results were described in percentage.

### 2.4. Thermal Properties Analysis of the Refined Tiger Nut Oil

A total of 1 L of the refined tiger nut oil was placed in a deep fryer. Constant high-temperature heating was performed at 150, 170, 190 and 210 °C, respectively, and samples were taken at 2, 4, 6, 8 and 10 h. 

#### 2.4.1. Physicochemical Properties

The AOCA (2012) method was used to determine the physicochemical indices (acid value (AV), iodine value (IV) and peroxide value (PV)) of tiger nut oil samples in every refining stage.

#### 2.4.2. Carbonyl Value

The determination of carbonyl value was according to the method of Chen et al. [22] with some modification.

The carbonyl compound in the tiger nut oil was reacted with 2, 4-dinitrophenylhydrazine to form 2, 4-dinitrophenylhydrazone, which appeared red in a basic solution. The absorbance was measured at 420 nm and the carbonyl value was calculated. The calculation formula was as follows:

(4) $$\mathrm{Carbonyl}\mathrm{value}\;(\mathrm{meq}/\mathrm{kg})=\frac{A}{1545\times M\times V_{1}\times V_{2}}\times 1000$$

where *A* is the measurement of the absorbance of the sample liquid; *M* is the sample weight, g; *V*_1_ is the total volume of the sample after dilution, mL; *V*_2_ is the volume of diluent for determination of the reagent, mL; 1545 is the average mg equivalent absorption coefficient of various carbonyl compounds.

#### 2.4.3. Polar Components

The content of polar components was measured 3 times every 2 h by the edible oil quality rapid detection instrument (equipment model: 892, Switzerland Vantone China Co., Ltd. (Hong Kong, China)).

### 2.5. Statistical Analysis

All experiments were conducted in triplicate, and the experimental data analysis and statistical significance were performed by Statistical Package for Social Science (SPSS) software package 22.0. Duncan’s test was used to assess the significance of the data at a significance level of *p* < 0.05. The diagrams were drawn out according to the data using Origin 8.5.

## 3. Results and Discussion

### 3.1. Changes in the Oil Yield and Oil Loss Rate of Refined Tiger Nut Oil

**Figure 1:**
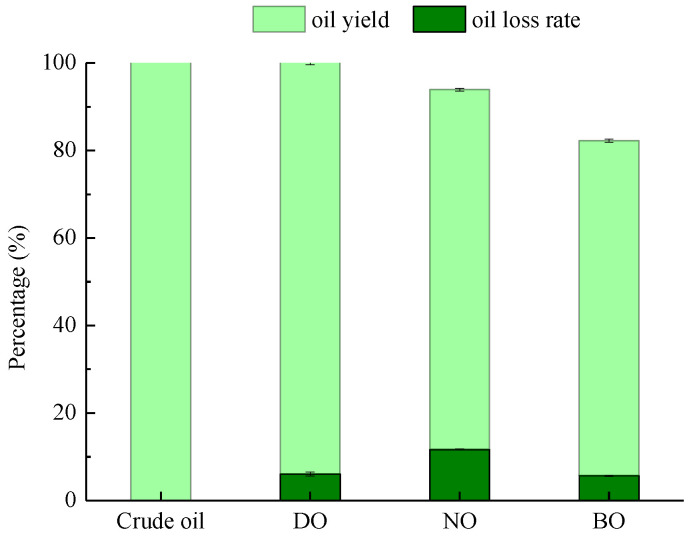
The oil yield and oil loss rate of the tiger nut oil in different refining processes.

Cold-pressed tiger nut oil was refined successively by degumming, neutralization and bleaching processes, and the yield and loss rate of the oil samples were measured and are shown in Figure 1. The loss rate of the degummed tiger nut oil was 6.07% after citric acid-assisted hydration degumming, indicating that phospholipids and other gelatinous impurities were effectively removed through the degumming process. Then, alkali refining was used to remove the free fatty acids (FFAs) from the degummed tiger nut oil, and the results showed that the neutralization samples treated with alkali refining exhibited a higher loss rate than other refining processes. The FFAs were neutralized in the process of alkali deacidification, and the pigments and phospholipids were adsorbed, and the embedding of neutral oil by soap was conducted [23]. On the whole, alkali deacidification had both degumming and bleaching effects on the oil. The loss rate of decolorization was 5.67%. This was due to the fact that in addition to pigment, saponin and phospholipid colloid could also be adsorbed by activated earth [24].

### 3.2. Sensory Color

**Figure 2:**
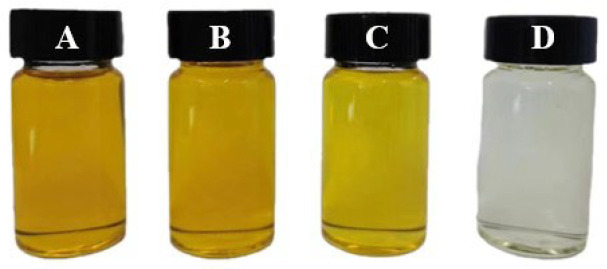
The sensory color of the tiger nut oil in refining processes. A: crude tiger nut oil, B: degummed tiger nut oil, C: neutralized tiger nut oil, D: bleached tiger nut oil.

Crude tiger nut oil contained various natural color substances [25]. Although the pigments contained in the oil were non-toxic, these would affect the appearance of the oil, so it needed to be bleached to meet consumer choices somewhat. The color changes of the tiger nut oil in different refining stages are shown in Figure 2. The color of crude oil was dark and with a lack of brightness, while the refined oil had higher brightness and transparency. The team’s previous experiment results indicated that the color difference value (ΔE*) after refining was 64.63. When ΔE* was greater than 3, obvious color changes could be seen by the naked eye, and the color of the oil was also significantly improved during the refining processes. In current research, all the oil samples were tested positive for a sensory color, indicating a tendency toward yellow tones. The bleaching process especially was the most important step for removing color completely due to activated earth. These results obtained were consistent with the results of Wu et al. [26].

### 3.3. Volatile Compounds and Clustering Analyses of Refined Tiger Nut Oil

**Figure 3:**
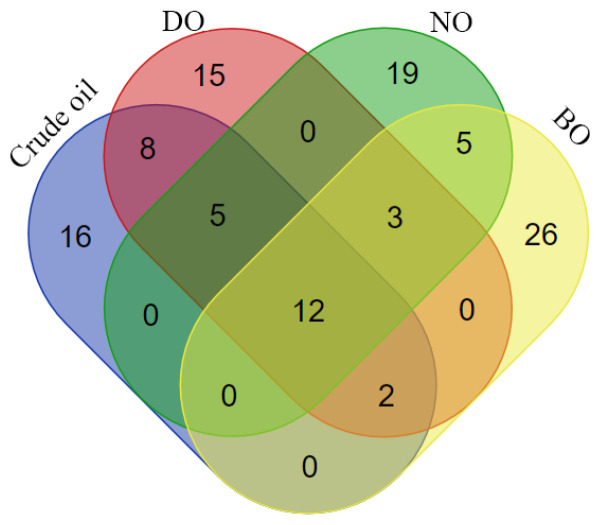
Total number and numbers of common and differential volatile compounds of different refined oils.

**Figure 4:**
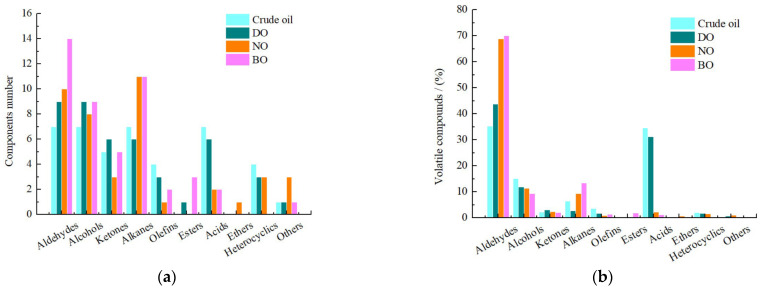
Variety and content changes of volatile compounds in the tiger nut oil during refining processes. (**a**) Component number, (**b**) volatile compounds.

Volatile components played a leading role in consumers’ choices of vegetable oils and were particularly important for their flavor [17]. We found several characteristic flavor volatiles detected in tiger nut oil after refining processes, which were consistent with results by matching information in the Agilent NIST 2.2 library. The total number of volatile compounds were as follows: 43 in crude lipids, 44 in degumming oil, 43 in neutralized oil and 47 in bleached oil. In all the tiger nut oil samples, there were 12 volatile compounds and different amounts of unique volatile components (Figure 3). A total of 109 volatile compounds were identified in four tiger nut oil samples, including 16 aldehydes, 19 alcohols, 13 ketones, 28 alkanes, 8 alkenes, 4 esters, 7 acids, 1 ester, 6 heterocycles and 6 other components, as shown in Figure 4a. 

As can be seen from Figure 4, the species of aldehydes in the volatile compounds of the four oil samples was significantly higher than that of the other substances. The relative contents of aldehydes in the NO and BO were more, which were 68.93% and 70.10%, respectively. In addition, although the number of alkanes were more than that of aldehydes, the proportion of alkanes was lower than that of aldehydes. No esters were detected in crude oil, degummed oil and bleached oil. Alcohols, ketones, olefins and heterocycles had lower content and the less species, which were detected during refining processes. The results showed that the refining processes had great influence on the species and contents of volatile compounds in the oil.

Aldehydes are general in the volatile flavor of vegetable oil, mainly produced by the automatic oxidation reaction of unsaturated fatty acids (UFAs) in the production and storage process and the lipid oxygenase pathway. Most aldehydes have better flavor and play a positive contribution to the flavor of vegetable oil. Aldehydes are derived primarily from the cleavage of lipid molecules by free radicals [27]. The peroxides generated by the automatic oxidation of lipids are easily decomposed when the temperature is more than 150 °C to generate alkoxy radicals and hydroxyl radicals, which are further cleaved to form volatile aldehydes, olefins, alcohols and other organic compounds [28]. Aldehydes have aroma characteristics such as fragrance odor, fruity odor and fatty odor, and their detected concentration is higher and their aroma threshold is lower, which are the main aromatic substances in the tiger nut oil. Hexanal is produced by the oxidation of linoleic acid, while octanal and nonanal are derivatives of oleic acid [29]. The results were consistent with the determination of fatty acid composition, which showed more oleic acid and linoleic acid in the tiger nut oil [5]. In the results, the great changes in the content of aldehydes might be related to the heat treatment in the refining process. Aldehydes were usually derived from the thermal oxidation degradation of UFAs. 

Alcohols are important precursors for the generation of long-chain esters, and most alcohols have higher threshold values, with less contributions to the aroma of the oil [30]. Unsaturated alcohols have a low threshold and contribute significantly to the flavor of the tiger nut oil, such as 2-Cyclohexen-1-ol. Most heterocyclic compounds have a low aroma threshold, which are mainly produced by the Maillard reaction. The results of tetramethyl-pyrazine and 2-pentyl-Furan showed the most content, which were generally produced by the oxidation of carbohydrates or UFAs. They had the flavor of nut odor, wood odor and butter odor, respectively, and the low content lead to less contributions to the flavor of the tiger nut oil. No heterocyclic compounds were detected in the bleached oil.

During the refining processes, the concentration of caproic acid, heptanoic acid, caprylic acid and pelanoic acid in the acids decreased significantly during the neutralization process, which reduced the irritating sour taste, sweat taste and spoilage odor. With the refining process, the concentration of alcohols increased first and then decreased. These alcohols contributed to the green color, fruit and leaven odor. Esters such as γ-valerolactone, which provided sweetness and a coconut odor, increased significantly during the bleaching process. All these changes would affect the flavor quality and consumer acceptance of the refined tiger nut oil [31].

**Figure 5:**
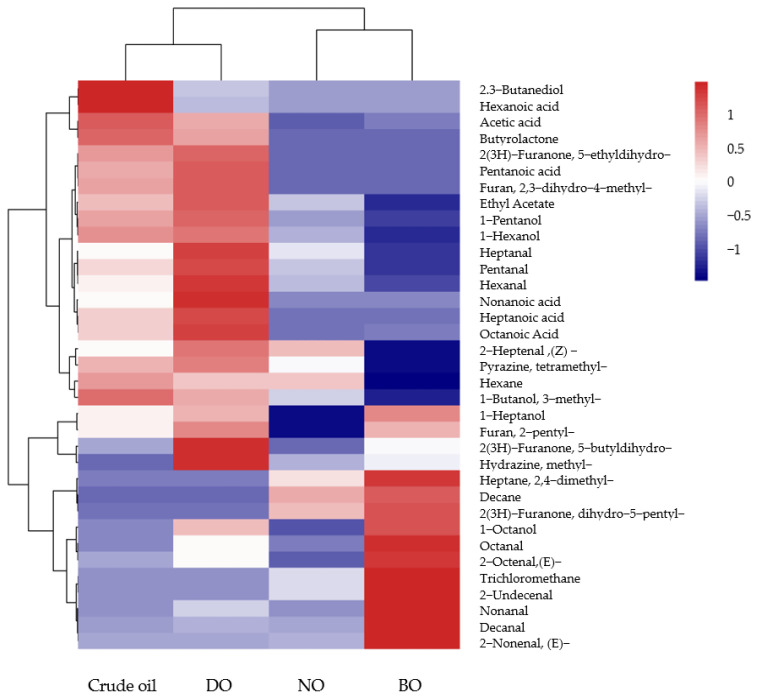
Clustering heat map of volatile compounds in refined tiger nut oils.

In order to more intuitively show the difference in the volatile compounds of the tiger nut oil obtained from the different refining processes, the content of the main volatile compounds was analyzed, and the results are shown in Figure 5. The content of the same volatile compound in different refined oil samples was standardized, and the color intensity varied from the maximum value (red) to the minimum value (blue), which was consistent with the content distribution of volatile compounds. In the figure, X cluster analysis reflected the overall similarity between different refined oil samples, and Y cluster analysis reflected the similarity between the distribution of volatile compounds. It could be seen from the X cluster analysis that according to the distribution of volatile compounds, the crude oil and the three different refined tiger nut oils could be divided into two categories, among which the crude oil and degummed oil were one category, and the neutralized oil and bleached oil were the other category. It could be seen from the Y-cluster analysis that the volatile compounds in different refining processes had their characteristic volatile components.

In conclusion, the results showed that a significant difference was found in the volatile constituents among the tiger nut oil during refining processes. Flavor contributors interacted with each other and then comprehensively presented the unique flavor of the tiger nut oil.

### 3.4. Physicochemical Property Analyses of Refined Tiger Nut Oil During High-Temperature Treatment

Acid value (AV) can reflect the degree of hydrolysis and deterioration of the edible oil [32]. The influence of heating treatment on the acid value of the refined tiger nut oil was shown in Table 2. With the heating process, the acid value of the oil increased from 0.14 mg/g to 0.561, 0.685, 1.682 and 2.306 mg/g at 150, 170, 190 and 210 °C, respectively, and the higher the heating temperature, the faster the AV of oil increased. With the high-temperature treatment, the rancidity of the oil was accelerated. This was because the oxidation reaction in the heating process generated hydroperoxides, which were unstable and produced free fatty acids [33]. GB 2716-2018 [34] (China’s National Standard Vegetable Oil for Food Safety) stipulates that the acid value of the edible vegetable oil during frying should be ≤5 mg/g. After continuous heating at 210 °C for 10 h, the maximum acid value of the oil was 2.306 mg/g, and less free fatty acids were produced, which almost had no impact on the quality of the oil. In addition, the acid value of oil during high-temperature treatment was also related to antioxidants. There was no decreasing trend within 10 h, indicating that the oxidation reaction was not complete, which might be due to the rich natural antioxidants such as V_E_ and sterol in the tiger nut oil [35].

It can be seen from Table 2, with heating at 150, 170, 190 and 210 °C, the iodine value (IV) of oil decreased from 79.085 g/100 g to 74.190, 72.972, 68.008 and 66.202 g/100 g, respectively. High-temperature treatment could break down the nutritional value of the tiger nut oil and accelerate the rancidity. This was due to the fact that the oil was oxidized and polymerized during heating treatment, and the cyclic polymer, glycerol ester dimer and other components were formed. Tiger nut oil is rich in oleic acid and linoleic acid [36]. And the fatty acids involved in heating hydrolysis were mainly linoleic acid, and their double bonds were oxidized to reduce their unsaturated bonds, leading to the formation of trans fatty acids and other harmful compounds [37]. However, monounsaturated fatty acids exemplified by oleic acid (C18:1) exhibited a higher resistance to oxidation [38]. Oleic acid is a stable monounsaturated fatty acid; the hydrogen atom in the allyl group is not active and not easy to react.

The peroxide value (PV) is an important index to evaluate the oxidation rate of oil, and it is also the comprehensive effect of oil oxidation and oxide decomposition [39,40]. With the heating treatment carried out at 150, 170, 190 and 210 °C, the peroxide value of the oil increased by 4.2, 7.6, 18.5 and 31.0 times, respectively (Table 2). An excessive peroxidation value of oil would produce an unpleasant odor and reduced palatability, and it destroyed the quality of oil [41]. GB 2716-2018 stipulates that the peroxide value of the edible vegetable oil is ≤0.25 g/100 g, and the maximum peroxide value of the tiger nut oil after heating at 170 °C for 10 h was 0.243 g/100 g. Although it did not exceed the national standard (GB 2716-2018) limit, it was not recommended to continuously heat the oil at high temperature. Generally, the peroxidation value was not used as an index to evaluate the oxidation degree of the oil alone, but to evaluate the deterioration degree together with other indexes.

### 3.5. Carbonyl Value of Refined Tiger Nut Oil During High-Temperature Treatment

**Figure 6:**
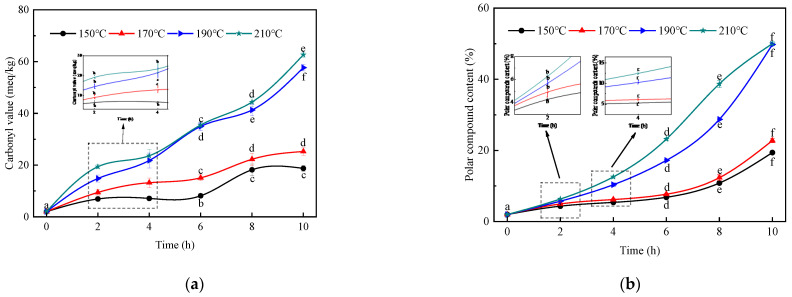
The thermal characteristics of the refined tiger nut oil. (**a**): carbonyl value, (**b**): polar compound content. Different letters (a–f) in the same column are significantly different (*p* < 0.05).

The carbonyl value can characterize the content of peroxides generated by oil oxidation which are further decomposed into fatty acids or glycerides and their polymers containing carbonyl and ketone groups, and its value can reflect the content of products of oil oxidation rancidity (carbonyl compounds such as ketones and aldehydes) and the degree of deterioration of the oil [22]. In Figure 6a, the carbonyl value of the refined tiger nut oil increased from 2.02 meq/kg to 18.73, 25.26, 57.66 and 62.63 meq/kg, with the extension of heating time at 150, 170, 190 and 210 °C, respectively. In addition, the carbonyl value also increased with the increase in temperature, which was consistent with Hu’s research results [42]. At the same time, according to chinese national standard (GB 5009.230-2016), the carbonyl value should not exceed 20 meq/kg, and the carbonyl value of 170 °C 8~10 h, 190 °C 4~10 h and 210 °C 4~10 h exceeded the national standard. Dicarbonyl compounds are not only important precursors of food flavor and color substances, but also precursors of heat processing hazards [43,44]. Therefore, it was recommended that the temperature should not be too high when the refined tiger nut oil was used as the frying oil, and the heating time should not be too long, in order to avoid the production and accumulation of harmful substances, which are harmful to human health, such as by causing gastrointestinal diseases and nervous system damage.

### 3.6. Total Polar Compound Percentages of Refined Tiger Nut Oil During High-Temperature Treatment

Polar components can be divided into three types according to the reaction type: triglyceride hydrolysate, oxidized triglyceride monomer and oxidized triglyceride polymerization product. The amount of these components with greater polarity than glyceryl ester is an important index to evaluate the quality deterioration of the frying oil [45]. With deep frying, peroxides and hydrogen peroxides produced by the oxidation of triglycerides were further decomposed into short-chain acids, aldehydes, ketones, alcohols and non-volatile products. 

As shown in Figure 6b, at 150, 170, 190 and 210 °C, with the extension of heating time, the polar compound content of the refined tiger nut oil rose from the initial value of 2.00% to 19.40%, 22.77%, 49.77% and 50.00%, respectively (50.00% is the instrument limit value). The content of polar compounds increased with the increase in the temperature, which might be related to the fact that the oil was exposed to high temperatures during the heating process with air or water, and a series of reactions such as polymerization, oxidation and hydrolysis reactions would occur, resulting in a series of volatile and non-volatile decomposition products [46]. GB 2716-2018 stipulates that the limit of polar components in frying oil is 27%, which can be used as a time-warning point for substandard frying quality. The United Kingdom, Japan and other countries stipulate that the total polar component of frying oil should not exceed 25%. When heated at 190 °C and 210 °C for 8 h, the content of polar compounds exceeded the national standard. Therefore, rational use of the early warning point of the oil frying time and strict control of heating time are beneficial to the frying quality of the refined tiger nut oil.

Therefore, by combining the requirements of the acid value, carbonyl value and polar compounds of the national vegetable oil health standard, the optimal heat treatment time of the oil was analyzed. The results showed that the heating temperature of the refined tiger nut oil was controlled at 150 °C, the heating time was controlled within 4 h or the heating temperature was controlled at 170 °C and the heating time was controlled within 2 h. This could better retain the quality of the refined tiger nut oil.

The oil that reached the waste point has continued to be used repeatedly under high-temperature conditions, which will aggravate harm to human health. The regulations of frying oil in our country mainly focus on acid value, carbonyl value, polar components and so on. It was reported that the acid value and carbonyl value of different vegetable oils in the frying process had a linear relationship with the total polar compound percentages [47], and the carbonyl value could be used to predict whether the total polar compound content exceeded the national standard and then judged regarding whether the oil had reached the warning point. However, due to the volatilization of free fatty acids and small molecular carbonyl substances, some studies also concluded that the acid value and carbonyl value could not be used as accurate indexes to evaluate oil quality [48].

## 4. Conclusions

The refining process conditions of degumming, alkali refining and bleaching the crude oil extracted from tiger nut (*Cyperus esculentus* L.) in China were optimized to obtain an edible functional oil. Tiger nut oil contained a high level of oleic acid, along with linoleic acid. The composition and relative content of the volatile compounds of the tiger nut oil in different refining processes were significantly different. The volatile aroma compounds of the degummed oil sample were aldehydes and acids, and the flavor components of samples obtained after neutralization and bleaching were aldehydes and alcohols and alkanes. Taken together, the findings highlighted the varying degrees of thermal stability among different high-temperature conditions. After prolonged heating at 150 °C and 170 °C for 10 h, the variations in the acid value and peroxide value were within the recommended value, indicating that the tiger nut oil was suitable for high-temperature heating as frying oil, but considering the change in the iodine value, carbonyl value and total polar compound percentages, it was not recommended to use the tiger nut oil with prolonged heating with high temperature. In the future, research will focus on the changes in the health indicators and nutritional components of the tiger nut oil in the frying process, the detection of the formation of harmful substances and the extension of the frying life of the oil, which is conducive to protect the health of consumers.

## Figures and Tables

**Table 1:** The conditions used in the different refining processes.

Varieties	Time (min)	Temperature (°C)	Others (*w*/*w*)
Degumming	30	40	Citric acid concentration (45%) Citric acid (0.25%), distilled water (3%)
Neutralization	20	60	NaOH solution (8%)
Bleaching	30	60	Activated earth (5%)

**Table 2:** The effect of high-temperature treatment on the physicochemical properties of the refined tiger nut oil.

Varieties	AV (KOH)/(mg/g)			
	150 °C	170 °C	190 °C	210 °C
0 h	0.138 ± 0.004 ^a^	0.138 ± 0.004 ^a^	0.138 ± 0.004 ^a^	0.138 ± 0.004 ^a^
2 h	0.163 ± 0.002 ^b^	0.180 ± 0.006 ^b^	0.205 ± 0.010 ^b^	0.278 ± 0.012 ^b^
4 h	0.189 ± 0.008 ^c^	0.223 ± 0.014 ^c^	0.347 ± 0.006 ^c^	0.384 ± 0.012 ^c^
6 h	0.208 ± 0.007 ^d^	0.330 ± 0.003 ^d^	0.572 ± 0.010 ^d^	0.640 ± 0.010 ^d^
8 h	0.313 ± 0.014 ^e^	0.439 ± 0.028 ^e^	0.974 ± 0.028 ^e^	1.223 ± 0.023 ^e^
10 h	0.561 ± 0.012 ^f^	0.685 ± 0.007 ^f^	1.682 ± 0.010 ^f^	2.306 ± 0.018 ^f^
IV (g/100 g)				
0 h	79.085 ± 1.366 ^a^	79.085 ± 1.366 ^a^	79.085 ± 1.366 ^a^	79.085 ± 1.366 ^a^
2 h	78.733 ± 0.183 ^ab^	78.298 ± 0.303 ^ab^	75.315 ± 0.328 ^b^	74.678 ± 0.272 ^b^
4 h	77.686 ± 0.127 ^bc^	77.373 ± 0.166 ^bc^	74.318 ± 0.174 ^bc^	73.840 ± 0.113 ^b^
6 h	77.264 ± 0.228 ^cd^	76.431 ± 0.341 ^cd^	74.041 ± 0.028 ^c^	73.464 ± 0.340 ^b^
8 h	76.393 ± 0.111 ^d^	75.786 ± 0.049 ^d^	73.575 ± 0.339 ^c^	71.834 ± 0.855 ^c^
10 h	74.190 ± 0.343 ^e^	72.972 ± 0.348 ^e^	68.008 ± 0.472 ^d^	66.202 ± 0.715 ^d^
PV (g/100 g)				
0 h	0.032 ± 0.002 ^a^	0.032 ± 0.002 ^a^	0.032 ± 0.002 ^a^	0.032 ± 0.002 ^a^
2 h	0.039 ± 0.001 ^ab^	0.049 ± 0.002 ^b^	0.063 ± 0.011 ^ab^	0.114 ± 0.006 ^b^
4 h	0.044 ± 0.001 ^b^	0.057 ± 0.000 ^c^	0.114 ± 0.000 ^ab^	0.161 ± 0.007 ^c^
6 h	0.063 ± 0.003 ^c^	0.088 ± 0.009 ^d^	0.137 ± 0.000 ^b^	0.221 ± 0.003 ^d^
8 h	0.084 ± 0.001 ^d^	0.161 ± 0.002 ^e^	0.229 ± 0.041 ^c^	0.410 ± 0.002 ^e^
10 h	0.133 ± 0.008 ^e^	0.243 ± 0.002 ^f^	0.592 ± 0.054 ^d^	0.993 ± 0.011 ^f^

Data are mean ± SD of three replicates. Different letters (a–f) in the same column are significantly different (*p* < 0.05).

## Data Availability

The data presented in this study are available on request from the corresponding author. The data are not publicly available due to privacy.
